# In-service nurse mentoring in 2020, the year of the nurse and the midwife: learning from Bihar, India

**DOI:** 10.1080/16549716.2020.1823101

**Published:** 2020-10-07

**Authors:** Adam D. Koon, Jerilyn Hoover, Sunil Sonthalia, Erica Rosser, Aboli Gore, Krishna D. Rao

**Affiliations:** a Department of International Health, Johns Hopkins Bloomberg School of Public Health, Baltimore, MD, USA; b Bihar Technical Support Program, CARE India, Patna, India

**Keywords:** Human resources for health, mentoring, quality of care, rural health, India

## Abstract

In-service nurse mentoring is increasingly seen as a way to strengthen the quality of health care in rural areas, where healthworkers are scarce. Despite this, the evidence base for designing large-scale programs remains relatively thin. In this capacity-building article, we reflect on the limited evidence that exists and introduce features of the world’s largest program, run by CARE-India since 2015. Detail on the mechanics of large-scale programs is often missing from empirical research studies, but is a crucial aspect of organizational learning and development. Moreover, by focusing on the complex ways in which capacity-building is being institutionalized through an embedded model of in-service mentorship, this article bridges research and practice. We point to a number of areas that require further research as well as considerations for program managers designing comparable workforce strengthening programs. With careful planning and cross-national policy learning, we propose that in-service nurse mentoring may offer a cost-effective and appropriate workforce development approach in a variety of settings.

## Background

The World Health Assembly recently designated 2020 as the ‘Year of the Nurse and the Midwife’, to commemorate the birth of nursing pioneer Florence Nightingale [1]. Because nurses and midwives are central to health service delivery, this campaign represents an overdue window of opportunity to increase the visibility of challenges within the health workforce [2]. Moreover, the emergence of Severe Acute Respiratory Syndrome Coronavirus 2 (SARS-CoV-2), has reinforced the essential role nurses and midwives play in responding to community health crises [3]. One challenge practitioners and researchers strengthening nursing and midwifery programs are likely to encounter however, is that empirical research on large-scale programs to support nurses is in relatively short supply [4].

Elsewhere, we recently reviewed the literature on in-service nurse mentoring as one potential strategy to improve clinical practice and retention [5]. We found 69 studies in 11 different countries, most of which were published since 2010, indicating that this is a growing area of interest. The majority of the studies were in rural settings, emphasizing that nurse mentoring is seen as a strategy to strengthen the quality of care in rural areas. Although the current literature about in-service nurse mentoring is primarily represented by several small-scale programs in high-income countries, we were surprised to learn that the largest nurse mentoring programs in the world (by a significant margin) were in low- and middle-income countries. We were also struck by the lack of detail in many of the research studies, which did not specify key features of programs, such as mentor to mentee ratios, frequency of contact, clinical content, or duration of mentoring. For this reason, we argue that taking a closer look at the experience of one in-service nurse mentoring program, where this information is available, would prove useful for capacity-building programs in a variety of settings.

In the following paper, we provide the programmatic detail of large-scale in-service nurse mentoring that is lacking in much of the peer-reviewed literature and is too difficult to represent within the narrow confines of a review article. The purpose of this ‘capacity-building’ article is to share our collective (and ongoing) experience in implementing and evaluating the world’s largest program. This is particularly important as large-scale programs present different kinds of challenges to public health practitioners and program managers [6]. In so doing, we point to a number of areas for further research and suggest implications for others designing comparable programs.

## Mentoring in nursing and midwifery

Nurses and midwives are essential for health systems to achieve universal health coverage [7]. Nurses and midwives are the largest segment of the health workforce, but also represent more than 50% (9 of the 17.4 million) of the global shortage of healthworkers [8]. This is particularly troubling as nurses and midwives perform a complex array of managerial and clinical tasks. They serve as first responders during complex crises and disasters, community advocates, coordinators within teams, and providers of basic primary care in underserved communities [4,8]. Moreover, their role is likely to expand as researchers continue to explore ways in which other tasks can be shifted from physicians to nurses [9].

The role of nurses and midwives in maternal, child, and newborn health is particularly well-established. Midwives can provide 87% of the essential care for women and newborns [10], with the potential to avert 83% of maternal deaths, stillbirths, and neonatal deaths worldwide [11]. Furthermore, as the burden of disease globally shifts towards noncommunicable diseases, nurses and midwives are increasingly seen as an important conduit for illness prevention, management, and treatment in community settings [8]. Thus, augmenting the scope of nurses and midwives in maternal and newborn health provides a cost-effective pathway to improve health outcomes, particularly in rural areas, where the need is greatest.

Nevertheless, there are several challenges in strengthening nursing and midwifery. In some countries, this includes demographic shifts in the workforce, compounded by shortages of physicians, retirement of registered nurses, and regulatory changes to the scope of clinical practice [12]. Redressing rural-urban workforce imbalances in nursing often focuses on increasing the supply of advanced practice nurses as opposed to enhancing the capabilities of nurses and midwives [13]. This is further complicated by the fact that nursing is a profession particularly prone to burnout and turnover [14]. Previous research has demonstrated, for example, that job satisfaction and turnover in rural hospitals has been associated with dissatisfaction with the work environment, including scarce professional development and educational opportunities in rural areas [15]. Thus, while increasing the supply of nurses and midwives should remain a priority, there is also a need to develop cost-effective strategies to nurture their abilities in rural settings, enhancing care and facilitating professional growth.

Consistent support to health professionals can be offered through training, coaching, and mentoring. Often training is provided in conjunction with clinical education, through preceptorships or other structured programs that transition students to practice or enhance the skills of existing practitioners [16]. Coaching is an interactive strategy that allows instructors in training programs to direct trainees in ways that enhance a narrowly-defined set of clinical skills [17]. Mentoring on the other hand is a relationship-building process in which the goal is professional growth and development [18]. This approach is particularly well-suited for rural areas where nurses are often removed from the formal support provided by training institutions, including clinical instructors [19]. While presumably a great deal of mentorship takes place informally in health facilities, the structure, scope, and scale of programs designed specifically for in-service nurses remains unclear.

## Learning from international experience

It is against this backdrop that CARE India, a non-profit organization working closely with the Government of Bihar and supported by the Bill and Melinda Gates Foundation, has been implementing the world’s largest nurse mentoring program in public health care facilities in rural Bihar since 2015. This program, called *Apaat kalin Matritva evam Navjaat Sishu Tatparta* (AMANAT), was designed after a 2012–2014 pilot study in 80 facilities of the Integrated Family Health Initiative found that the clinical skills of auxiliary nurse midwives (ANMs) in Bihar were underdeveloped. In India, ANMs typically have a high school education plus a two-year diploma that provides training in preventive and promotive care, with six months of basic midwifery skills to conduct normal deliveries. AMANAT was an on-site in-service nurse mentoring program that focused on improving basic and emergency obstetric and newborn care (BEmONC) in 320 public sector facilities across the state of Bihar. The first phase of the intervention ran from 2015 to 2017 and consisted of a four-stage staggered rollout, covering ANMs and staff nurses posted in the labor rooms of those facilities. On average, six to eight nurses (ANMs and staff nurses) from each facility were mentored by two nurse mentors. Each nurse mentor pair was responsible for four BEmONC facilities, where they would visit for a week every month. Over eight to nine months, these mentor pairs rotated weekly to cover one of four public sector facilities. Given the lack of adequately skilled nurses in Bihar, these mentors, with Bachelor’s degrees in nursing, were recruited from across India and underwent an Induction course of six weeks by CARE India-Bihar. This included technical sessions on maternal & newborn care, management of maternal and neonatal complications, Government of India protocols and guidelines, simulations, team building, communication, and debriefing skills. Refresher trainings were conducted for four days every three months.

Mentoring in facilities incorporated structured learning sessions with a mix of didactic instruction including basic nursing procedures, infection prevention, basic obstetric and neonatal practices, management of complications such as post- partum hemorrhage, birth asphyxia, pre-eclampsia (and others), documentation and reporting, team rapport and communication. This was structured into weekly mentoring sessions once a month, with interpersonal and team-building skills first, and involved hands-on mentoring throughout the course of service delivery. Hands-on mentoring while interacting with patients was the core of on-site program activities. In the absence of these cases during the mentoring days, normal and complicated deliveries were simulated using the PRONTO Pack simulation kits, which included a birth simulator (MamaNatalie) worn by a demonstrator to resemble a pregnant woman. Evaluations of this interactive training have demonstrated its success (INSERT ref). Hands-on guidance involved nurse–mentors working alongside mentees, observing them and co-managing cases when necessary. While the package of interventions was standardized, mentoring interactions were tailored to accommodate facility-specific needs, a critical component of the interpersonal mentoring process.

Rigorous program evaluations were conducted to assess the impact of AMANAT. In a pre-post test comparison, proportional (percentage point) post interventional increases in correct actions taken by ANMs were 17.5 (95% CI 14.8 to 20.2) for managing normal deliveries, 25.9 (95% CI 22.4 to 29.4) for postpartum hemorrhage, and 28.4 (95% CI 23.2 to 33.7) for neonatal resuscitation [6]. Another similar evaluation revealed that the impact was mostly sustained, with correct intrapartum practices during normal delivery slightly changing from 44.2% (95% CI: 42.1 to 46.4) when mentoring in the last three months to 39.1% (37.7–40.5) one year post-mentoring [20]. These findings suggest that large-scale in-service mentoring can improve the performance of nurse midwives who have only a basic level of pre-service clinical training. Because the first phase of AMANAT showed potential, the findings from these evaluations were used to design the next iteration of the program, AMANAT-Jyoti, which focuses more extensively on health systems strengthening.

**Figure 1.**
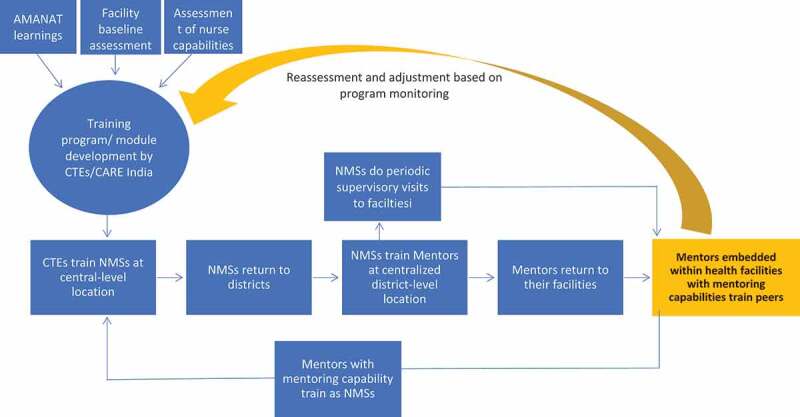
Structure of AMANAT-Jyoti.

This second phase of the nurse mentoring program, AMANAT-Jyoti, involves a more elaborate, and potentially sustainable, mentoring structure. See Table 1 for program characteristics. Mentorship begins with Clinical Training Experts, who support Nurse Mentor Supervisors that rotate through a set coverage area in the same way as the previous phase’s nurse mentors. In AMANAT-Jyoti, two mentors from each public facility (often previously high performing mentees) are trained and supported to mentor six to eight mentees in the same facility, with additional support from the Nurse Mentor Supervisors. While evaluations are ongoing, this structure holds the promise to provide consistent endogenous support in ways that the previous phase of AMANAT did not. Mentors are peers and work side-by-side with mentees on a daily basis while receiving regular support from Nurse Mentor Supervisors as well as Masters-level Clinical Training Experts. See Figure 1 for organizational structure.

**Table 1.** AMANAT-Jyoti characteristics.

	Number (N)
***AJ – participants (total trained)***	
CTEs	10
NMSs	60
Mentors	721
Mentees	3217
***Scale of Facility-Based Mentoring***	
Facility	361/516 in Bihar
Blocks	361/534
Districts	33/36
**Frequency of Contact**	
CTEs to NMSs	Every day (Physical support 2–3 days/week, Virtually present everyday)
NMS to Mentors	6 days in 2–3 months physically virtually everyday
Mentors to Mentees	Everyday (Atleast one mentee and mentor interaction in each facility)
**Frequency of Trainings**	
NMS	Quaterly and whenever needed
Mentors	Rigorous Facilitator training 6 weeks in a year average 3 weeks of 6 month interval. On site support 6 days in 2–3 month time. And whenever needed
Mentees	3 days structure training in 2–3 weeks interval, unstructured capacity building throughout as mentor present in the same facility.

While AMANAT and AMANAT-Jyoti show promise, they are not without their challenges. Both programs are resource-intensive and challenge a rapidly changing health system to provide community-level support at-scale. Also, the programs frequently encounter difficulties with retention, primarily due to the difficulty in coordinating transfers and postings with the Government of Bihar. Finally, as these programs mature, they naturally become more complex, potentially placing additional administrative demands on state governments to manage program operations through carefully sequenced donor transitions.

More research is needed to understand operationally how in-service nurse mentoring programs, such as AMANAT/AMANT-Jyoti, evolve and pedagogically how the gap between knowledge and practice among participants can be narrowed. Much remains unknown about the optimal ratio of mentors to mentees at various levels of training, a common gap in community health programming [21]. Similarly, how these mentors are supported and engaged in self-sustaining processes of continuous learning and professional development are unclear. Also, research on strategies to mitigate attenuation of newly acquired clinical skills as practitioners transition to more autonomous work environments is needed. Linkages between knowledge, competency, and performance (know-do gaps) remain poorly understood and are increasingly seen as impediments to effective service delivery at-scale [22]. Finally, the extent to which enhanced support can overcome systemic structural complications in resource-constrained settings is difficult to gauge and yet a key consideration for program managers in LMICs [23]. In this way, more mixed methods research within and building on the Indian experience can help strengthen models of in-service nurse mentoring at-scale.

Nevertheless, there is reason to think that some features of in-service nurse mentoring programs such as AMANAT-Jyoti might be successfully adapted to other contexts. While the Indian experience is focused on boosting the capacity of auxiliary nurse midwives with basic training, rural nurses and midwives in other settings may operate from a stronger clinical knowledge base and benefit from preceptorships which smooth the transition to practice. Also, connectivity and advances in telemedicine represent potential for consistent mentoring across vast geographic areas, which is much more difficult in rural India. In addition to this, county and state health departments in some countries benefit from greater management capacity and receive federal assistance through workforce strengthening programs. In these circumstances, health officials are able to recruit healthworkers from, and better target healthworkers in, rural communities. How in-service mentorship can be embedded and dovetail with existing initiatives should be explored in future implementation research; however, we argue that despite a nascent pool of evidence on in-service mentoring programs at-scale, the Indian experience shows that collective intersectoral action focused on capacity-building is possible.

## Conclusions

Healthcare in rural areas is urgently in need of new ideas to address worrying workforce and epidemiological trends, particularly with respect to health disparities. As a cost-effective segment of the health workforce, some argue that the importance of nurses and midwives is growing as we understand more about what makes for effective health systems [2]. While the evidence remains in its infancy, in-service nurse mentoring offers a fertile ground for future research. Moreover, this represents a potential platform for strengthening the quality of clinical care, particularly in rural primary care facilities, where formal training and professional development programs are scarce. In 2020, the year of the nurse and the midwife, finding new ways to effectively support and nurture them is both an intellectual and moral challenge for health systems that seek to make the world a healthier and fairer place to live.

## Paper context

This paper highlights an overlooked and under-researched approach to strengthening the capacity of healthworkers: in-service nurse mentoring. We recently found that this is an increasingly popular way of strengthening service delivery, and yet very little programmatic detail is included in research on these programs. Moreover, much remains unknown about how they work and how they can be improved. This paper shares lessons learned from our experiences working in the world’s largest in-service nurse mentoring program in India. We provide evidence-informed practical guidance to help future programs strengthen healthworker capacity in a variety of settings.
